# Epidemiology of Pediatric Transfusion Reactions

**DOI:** 10.1001/jamanetworkopen.2026.9274

**Published:** 2026-04-27

**Authors:** Elizabeth F. Stone, Daniel Chacreton, Alexandra Jimenez, Shannon Kelly, Ruchika Goel, Jeanne E. Hendrickson, Marianne E. Nellis, Martha Sola-Visner, Oliver Karam, Ravi M. Patel, Sarah Vossoughi, Madhav Vissa, Sarah Wilson, Jeffrey J. VanWormer, Sylvia Titi Singer, Sara Bakhtary, Elliott P. Vichinsky, Naomi L.C. Luban, Cassandra D. Josephson, Matthew S. Karafin

**Affiliations:** 1 Department of Pathology and Cell Biology, Columbia University Irving Medical Center, New York, New York; 2 Clinical Research Practice, Westat, Rockville, Maryland; 3 Department of Clinical Pathology and Laboratory Medicine, Weill Cornell Medical Center, New York, New York; 4 Department of Pediatrics, University of California San Francisco Benioff Children’s Hospital, Oakland; 5 Department of Internal Medicine, Simmons Cancer Institute, Southern Illinois University School of Medicine, Springfield; 6 Corporate Medical Affairs, Vitalant, Scottsdale, Arizona; 7 Department of Pathology, Johns Hopkins University School of Medicine, Baltimore, Maryland; 8 Department of Pathology and Laboratory Medicine, Center for Transfusion and Cellular Therapies, Emory University School of Medicine, Atlanta, Georgia; 9 Department of Laboratory Medicine, Yale University School of Medicine, New Haven, Connecticut; 10 Division of Critical Care Medicine, Department of Pediatrics, Weill Cornell Medicine, New York, New York; 11 Division of Newborn Medicine, Boston Children’s Hospital and Harvard Medical School, Boston, Massachusetts; 12 Pediatric Critical Care Medicine, Department of Pediatrics, Yale School of Medicine, New Haven, Connecticut; 13 Department of Pediatrics, Emory University School of Medicine and Children’s Healthcare of Atlanta, Atlanta, Georgia; 14 Department of Pathology, SUNY Downstate Health Sciences University, Brooklyn, New York; 15 Marshfield Clinic Research Institute, Marshfield, Wisconsin; 16 Hemoglobinopathy Reference Laboratory, Department of Hematology/Oncology, University of California San Francisco Benioff Children’s Hospital, Oakland; 17 Department of Laboratory Medicine, University of California San Francisco, San Francisco; 18 Division of Hematology, University of California San Francisco Benioff Children’s Hospital Oakland, Oakland; 19 Children’s National Research Institute, Washington, DC; 20 Department of Pediatrics, George Washington University School of Medicine and Health Sciences, Washington, DC; 21 Cancer and Blood Disorders Institute, Johns Hopkins All Children’s Hospital, St Petersburg, Florida; 22 Departments of Oncology, Pediatrics, and Pathology, Johns Hopkins University School of Medicine, Baltimore, Maryland; 23 Department of Pathology and Lab Medicine, University of North Carolina at Chapel Hill, Chapel Hill

## Abstract

**Importance:**

Pediatric hemovigilance is a nascent field in transfusion medicine. The lack of standardized hemovigilance reporting in the US makes it difficult to determine age-specific transfusion reaction rates and risks.

**Objective:**

To evaluate the rates and epidemiology of transfusion reactions reported to transfusion services in neonatal and pediatric populations.

**Design, Setting, and Participants:**

This cohort study analyzed transfusion reactions occurring in children younger than 18 years reported to 8 hospitals’ transfusion services during April 1, 2019, through December 31, 2023. Data were evaluated from March 2024 to June 2025 using standardized data collection forms and associated electronic health records.

**Exposure:**

Patients who received transfused blood products (red blood cells [RBCs], platelets, plasma, or cryoprecipitate) with at least 1 transfusion reaction reported to the transfusion service.

**Main Outcomes and Measures:**

Reaction rates per 100 000 products transfused were calculated. Pediatric transfusion reactions were characterized in detail, including reported severity and imputability; product type; patient age, sex, race, and ethnicity; and reported symptoms, premedication, and clinical management.

**Results:**

The sample included 228 886 products transfused to 22 628 patients (median [IQR] age, 4.2 [0.3-12.4] years; 127 903 males [55.9%]). The products were transfused to patients of Asian (18 649 [8.2%]), Black (37 673 [16.5%]), White (93 824 [41.0%]), multiracial (3680 [1.6%]), other (68 857 [30.1%]), or unknown race (6203 [2.7%]) and Hispanic or Latinx (52 398 [22.9%]), non-Hispanic and non-Latinx (144 017 [62.9%]), and unknown ethnicity (32 471 [14.2%]). A total of 1165 imputable transfusion reactions were reported, with an overall reaction rate of 0.52% (95% CI, 0.49%-0.55%). Patients aged 5 to 11 years had the highest reported transfusion reaction rate (891.11 [95% CI, 799.81-989.11] per 100 000 products transfused). Platelet transfusions had the highest transfusion reaction rates (821.75 [95% CI, 754.14-893.80] per 100 000 products transfused), with allergic reactions being most common (506.04 [95% CI 453.30, 563.24] per 100 000 products transfused), whereas RBC transfusions had more reported febrile nonhemolytic transfusion reactions (FNHTRs; 296.20 [267.06, 327.67] per 100 000 products transfused) than other types of reactions. The most common symptoms were urticaria (69.6% [368 of 529 patients]) in allergic reactions, fever (96.5% [559 of 579 patients]) in FNHTRs, and acute respiratory distress (87.5% [21 of 24 patients]) in transfusion-associated circulatory overload (TACO); the most common treatments were antihistamines (80.3% [425 of 529 patients]) for allergic reactions, antipyretics (67.9% [393 of 579 patients]) for FNHTRs, and diuretics (83.3% [20 of 24 patients]) for TACO. Many patients (35.8% [107 of 299]) did not receive premedication after the first reaction in subsequent transfusions, regardless of reaction type. When transfusion reactions recurred, they were often of the same type (77.9% of reactions [120 of 154] after allergic reactions were allergic; 72.1% of reactions [98 of 136] after FNHTRs were FNHTRs).

**Conclusions and Relevance:**

In this cohort study of pediatric transfusion reactions, reactions appeared to be age dependent, and rates of allergic reactions and FNHTRs were higher than rates from previously published, possibly underreported, predominantly adult data. These findings underscore the importance of pediatric-specific hemovigilance to improve recognition, reporting, and safety monitoring of transfusion reactions.

## Key Points

**Question:**

What are the rates and epidemiology of pediatric and neonatal transfusion reactions in the US?

**Findings:**

In this cohort study examining 228 886 transfusions administered to 22 628 pediatric patients across 8 US hospitals, the overall transfusion reaction rate was 0.51%. Allergic reactions and febrile nonhemolytic transfusion reactions occurred more often than in published all-age National Healthcare Safety Network data.

**Meaning:**

These findings suggest pediatric transfusion reactions occur more frequently compared with previously published data from predominantly adult patients, underscoring the importance of pediatric-specific hemovigilance to improve recognition, reporting, and safety monitoring.

## Teaser

This cohort study assesses the epidemiology and rates of transfusion reactions reported in US hospitals among pediatric patients.

## Introduction

Blood transfusion is the most common procedure for hospitalized patients.^1,2^ Current research suggests that pediatric patients may have a higher rate of transfusion reactions compared with adults, especially for febrile nonhemolytic transfusion reactions (FNHTRs) and allergic transfusion reactions.^3,4,5^ However, there is currently no required national hemovigilance system in the US. Consequently, current data on transfusion reactions are based on a variety of sources, including studies with disparate, variable, or nonstandardized reporting.^6,7,8,9,10,11^ The lack of standardization diminishes the value of any comparison between smaller institutional studies. To understand whether differences in transfusion reaction rates found between adult and pediatric transfusion recipients represent inherent physiological differences in the pediatric population, differences in clinical practices, or differences in recognition and reporting, a robust analysis of transfusion reactions in the US is necessary.

To address the current knowledge gaps regarding pediatric transfusion reactions, we designed a multicenter, prospective, observational, longitudinal cohort study to describe the epidemiology of transfusion reactions in children in the US compared with transfusion reactions in 2 previously published large studies.^4,7^ All reactions occurring after inpatient and outpatient pediatric transfusions reported to the hospital transfusion service from April 1, 2019, through December 31, 2023, from 8 participating hospitals, including 3 large tertiary children’s hospitals, were reviewed. The reaction incidence rate per 100 000 products transfused in the pediatric study population was calculated. We also examined transfusion reaction–associated outcomes and exposures, exploring variations in reaction rates stratified by key demographic and clinical features, including age, product type transfused, race, sex, and history of previous transfusion reactions.

This analysis provides the first standardized, multicenter evaluation of pediatric-specific transfusion reactions. Beyond quantifying the incidence of all pediatric transfusion reactions, findings from this study may inform targeted prevention strategies, improve recognition and reporting practices, and support the development of evidence-based guidelines to enhance transfusion safety for pediatric patients.

## Methods

Each participating hospital obtained institutional review board approval for participation in the REDS-IV-P overall contract. The single central institutional review board for REDS-IV-P determined this study met 45 CFR 46.104 (d) category (4iii) for exempt review and granted a waiver of informed consent because all data were deidentified. This study was structured to align with the Strengthening the Reporting of Observational Studies in Epidemiology (STROBE) guidelines.

### Study Design

This prospective cohort study used data from the Recipient Epidemiology and Donor Evaluation Study-IV-Pediatric (REDS-IV-P) vein-to-vein (V2V) database and data from a supplemental transfusion reaction portal (eFigure in Supplement 1). The transfusion reaction portal collected data on detailed signs and symptoms, premedication, and treatments for health care provider–reported transfusion reactions associated with pediatric patients. Multiple transfusion reactions could be reported for each transfusion when appropriate. Thus, depending on the analysis, we used 1 of 3 distinct methods for counting transfusion reactions: (1) counting all reactions individually, (2) counting transfusions with multiple reactions only once to analyze patient demographic characteristics, or (3) excluding transfusion reactions when multiple reactions were associated with a single transfusion to examine relevant symptoms and treatment-related outcomes. The reported reactions were classified based on imputability, in alignment with National Healthcare Safety Network (NHSN) Biovigilance Component Hemovigilance Module Surveillance Protocol case definitions.^12^ Additionally, the study used all-age acute and delayed transfusion reaction rate data reported from the NHSN Hemovigilance Module^7^ and a second pediatric cohort, with most pediatric transfusion reaction data from the Association for the Advancement of Blood and Biotherapies Center for Patient Safety,^4^ as comparison groups for our pediatric population.

The REDS-IV-P V2V database included information on blood transfusion donors, recipients, and components collected from 4 participating blood collection centers, along with academic and community hospitals. Participating study sites were located in California, Connecticut, Pennsylvania, Wisconsin, and New York. The study data collection period began on April 1, 2019, and ended on December 31, 2023; data were analyzed from March 2024 to June 2025. Patient characteristics (eg, sex, race, and ethnicity) were extracted from patient medical records in the REDS-IV-P V2V database. Missing or unrecorded categorical study data were recoded as unknown. Racial categories captured in the database included American Indian or Alaska Native, Asian, Black, Native Hawaiian or Other Pacific Islander, White, and other. The study included the following races, reported less frequently, in the other classification: American Indian or Alaska Native, Native Hawaiian or Other Pacific Islander, and other (other was not specified further). Ethnicities included Hispanic or Latinx and non-Hispanic and non-Latinx. Additionally, a multiracial classification was created to capture combined racial and ethnic groups, including Black and Hispanic or Latinx, Asian and Hispanic or Latinx, Native Hawaiian or Other Pacific Islander and Hispanic or Latinx, American Indian or Alaska Native and Hispanic or Latinx, and other nonspecified multiethnic groups. A detailed description of the study database has been previously published.^13^

### Study Population

The REDS-IV-P study population included pediatric patients (aged <18 years) who received a transfusion at a participating study site during the study period. Only transfusions associated with red blood cell (RBC), cryoprecipitate, plasma, and platelet products were considered eligible for inclusion in the study; whole-blood and apheresis granulocyte transfusions were excluded. Finally, reported reactions that did not meet either the definite, probable, or possible imputability criteria and those associated with individuals who were aged 18 years or older were excluded from the analysis.

### Statistical Analysis

The study included a descriptive analysis to summarize the study population demographics and reaction clinical outcomes. Comparative analyses were conducted using χ^2^ tests or Fisher exact tests for evaluating counts of categorical variables. When expected cell counts were less than 5 but greater than 1, the Yates continuity correction was applied to χ^2^ tests to reduce the risk of type I errors in these instances. Fisher exact tests were used when expected cell counts were less than 1. Reaction rates were calculated per 100 000 transfused products, with a Garwood exact 95% CI as implemented through the γ distribution for Poisson counts.^14^ Finally, relative risks with 95% CIs were calculated to complete the reaction rate comparisons. Statistical significance was defined as *P* < .05, and *P* value tests were 2 sided. Data cleaning and statistical analysis were performed using Python version 3.10.11 (Python Software Foundation). Of note, for variables that were considered unknown or undefined, no statistical tests were performed.

## Results

A total of 228 886 products were transfused in 22 628 pediatric patients (median [IQR] age, 4.2 [0.3-12.4] years; 127 903 males [55.9%] and 100 905 females [44.1%]) from 8 hospitals (7 academic medical centers, 1 community hospital) (Table 1). The products were transfused to patients of Asian (18 649 [8.2%]), Black (37 673 [16.5%]), White (93 824 [41.0%]), multiracial (3680 [1.6%]), other (68 857 [30.1%]), or unknown race (6203 [2.7%]) and Hispanic or Latinx (52 398 [22.9%]), non-Hispanic and non-Latinx (144 017 [62.9%]), and unknown ethnicity (32 471 [14.2%]). There were 1183 imputable (ie, definite, probable, or possible) transfusion reactions that occurred; of these, multiple reactions occurring during the transfusion of a single product were counted only once for analysis of patient demographic characteristics. Thus, 1165 imputable reactions were reported in 866 pediatric patients (rate, 0.51% [95% CI, 0.48%-0.54%]) (Table 1). The median (IQR) age of patients with transfusion reactions was 8.2 (4.0-13.6) years (*P* < .001). There were no significant differences observed by sex.

**Table 1.** Descriptive Summary of Study Population

Variable	Overall	No transfusion reaction	Transfusion reaction	*P* value
Total transfusions, No. (%)^a^	228 886 (100.0)	227 721 (99.5)	1165 (0.5)	NA
Unique patients receiving transfusions, No. (%)	22 628 (100.0)	21 762 (96.2)	866 (3.8)	NA
Age, median (IQR), y	4.2 (0.3-12.4)	4.2 (0.3-12.4)	8.2 (4.0-13.6)	<.001^b^
Weight, median (IQR), kg	16.3 (5.2-42.5)	16.2 (5.2-42.4)	26.5 (15.7-50.6)	<.001^b^
Sex, No. (%)				
Female	100 905 (44.1)	100 376 (44.1)	529 (45.4)	.37^c^
Male	127 903 (55.9)	127 267 (55.9)	636 (54.6)	.37^c^
Unknown^d^	78 (0.0)	78 (0.0)	NA	NA
Race, No. (%)^e^				
Asian	18 649 (8.2)	18 534 (8.1)	115 (9.9)	.05^c^
Black	37 673 (16.5)	37 523 (16.5)	150 (12.9)	<.001^c^
White	93 824 (41.0)	93 293 (41.0)	531 (45.6)	.005^c^
Multiracial	3680 (1.6)	3659 (1.6)	21 (1.8)	.64^c^
Other	68 857 (30.1)	68 524 (30.1)	333 (28.6)	.15^c^
Unknown^d^	6203 (2.7)	6188 (2.7)	15 (1.3)	NA
Ethnicity, No. (%)				
Hispanic or Latinx	52 398 (22.9)	52 094 (22.9)	304 (26.1)	.06^c^
Non-Hispanic and non-Latinx	144 017 (62.9)	143 281 (62.9)	736 (63.2)	.06^c^
Unknown^d^	32 471 (14.2)	32 346 (14.2)	125 (10.7)	NA
Hospital, No. (%)^f^				
1	72 637 (31.7)	72 262 (31.7)	375 (32.2)	.74^c^
2	17 233 (7.5)	17 160 (7.5)	73 (6.3)	.10^c^
3	32 574 (14.2)	32 402 (14.2)	172 (14.8)	.60^c^
4	9147 (4.0)	9102 (4.0)	45 (3.9)	.82^c^
5	16 808 (7.3)	16 705 (7.3)	103 (8.8)	.05^c^
6	41 613 (18.2)	41 366 (18.2)	247 (21.2)	.007^c^
7	37 001 (16.2)	36 867 (16.2)	134 (11.5)	<.001^c^
8	1873 (0.8)	1857 (0.8)	16 (1.4)	.03^c^

Abbreviation: NA, not applicable.

^a^ Transfusions associated with multiple reaction types are counted as 1 event.

^b^ Mann-Whiney *U* test used to compare medians for continuous variables.

^c^ Two-proportion *z* test used to compare proportions for categorical variables.

^d^ Unknown includes missing values and values that were not recorded.

^e^ The multiracial classification includes individuals who self-identify as American Indian or Alaska Native and Hispanic or Latinx, Asian and Hispanic or Latinx, Black and Hispanic or Latinx, Native Hawaiian or Other Pacific Islander and Hispanic or Latinx, or other nonspecified multiethnic groups. The other classification includes individuals who self-identify as American Indian or Alaska Native, Native Hawaiian or Other Pacific Islander, or other.

^f^ Hospital 8 is a community hospital. The remaining hospitals are academic medical centers.

### Rates of Transfusion Reactions in Pediatric Patients Compared With Patients of All Ages

Overall, pediatric blood transfusion recipients had higher rates of transfusion reactions compared with the all-ages data reported from the NHSN Hemovigilance Module^7^ and a separate pediatric cohort^4^ (Table 2). Per 100 000 products transfused, the imputable transfusion reaction rate was 516.85 (95% CI, 487.81-547.16) in our pediatric cohort compared with 219.49 (95% CI, 216.32-222.69) in the published NHSN all-ages cohort (*P* < .001) and 537.86 (95% CI, 510.07-566.77) in the separate pediatric cohort (*P* = .31). Pediatric patients also had higher rates per 100 000 products transfused of allergic reactions (236.36 [95% CI, 216.86-257.15] vs 91.93 [95% CI, 89.88-94.01]; *P* < .001) and FNHTRs (258.64 [95% CI, 238.23-280.34] vs 91.22 [95% CI, 89.18-93.29]; *P* < .001) compared with the NHSN all-ages cohort. Notably, pediatric patients had similar rates per 100 000 products transfused of transfusion-associated circulatory overload (TACO; 13.98 [95% CI, 9.56-19.74] vs 11.20 [95% CI, 10.49-11.94]; *P* = .22), transfusion-associated dyspnea (TAD; 6.12 [3.34-10.26] vs 4.06 [95% CI, 3.64-4.52]; *P* = .13), delayed hemolytic transfusion reactions (DHTRs; 0.87 [95% CI, 0.11-3.16] vs 3.12 [95% CI, 2.75-3.52]; *P* = .06), and transfusion-related acute lung injury (TRALI; 0.87 [95% CI, 0.11-3.16] vs 0.73 [95% CI, 0.56-0.94]; *P* = .69) compared with the NHSN all-ages cohort. Compared with our cohort, the separate pediatric cohort^4^ had a higher rate per 100 000 products transfused of allergic reactions (323.40 [95% CI, 301.94-345.99]; *P* < .001) but lower rates per 100 000 products transfused of FNHTRs (170.72 [95% CI, 155.22-187.34]; *P* < .001) and TACO (3.45 [95% CI, 1.58-6.55]; *P* < .001), while rates per 100 000 products transfused of TAD (5.37 [95% CI, 2.94-9.01]; *P* = .73), DHTRs (0.38 [95% CI, 0.01-2.14]; *P* = .60), and TRALI (1.53 [95% CI, 0.42-3.93]; *P* = .69) were similar.

**Table 2.** REDS-IV-P Acute and Delayed Transfusion Reaction Rate Comparisons

Adverse reaction	Reported event, No.	Definite, probable, or possible event, No. (%)^a^	Imputable rate per 100 000 products transfused (95% CI)	NHSN all-ages cohort rate per 100 000 products transfused (95% CI)^7^	REDS-IV-P vs NHSN all-ages cohort RR (95% CI)	NHSN all-ages cohort comparison *P* value	Separate pediatric cohort imputable rate per 100 000 products transfused (95% CI)^4^	REDS-IV-P vs separate pediatric cohort RR (95% CI)	Separate pediatric cohort comparison *P* value
All adverse reactions	1325	1183 (89.3)	516.85 (487.81-547.16)	219.49 (216.32-222.69)	2.35 (2.22-2.50)	<.001^b^	537.86 (510.07-566.77)	0.96 (0.89-1.04)	.31^b^
Allergic	550	541 (98.4)	236.36 (216.86-257.15)	91.93 (89.88-94.01)	2.57 (2.36-2.81)	<.001^b^	323.40 (301.94-345.99)	0.73 (0.66-0.81)	<.001^b^
FNHTR	722	592 (82.0)	258.64 (238.23-280.34)	91.22 (89.18-93.29)	2.84 (2.61-3.08)	<.001^b^	170.72 (155.22-187.34)	1.52 (1.34-1.71)	<.001^b^
TACO	32	32 (100.0)	13.98 (9.56-19.74)	11.20 (10.49-11.94)	1.25 (0.88-1.78)	.22^b^	3.45 (1.58-6.55)	4.05 (1.93-8.48)	<.001^b^
TAD	15	14 (93.3)	6.12 (3.34-10.26)	4.06 (3.64-4.52)	1.51 (0.88-2.57)	.13^b^	5.37 (2.94-9.01)	1.14 (0.54-2.39)	.73^b^
DHTR	4	2 (50.0)	0.87 (0.11-3.16)	3.12 (2.75-3.52)	0.28 (0.07-1.13)	.06^b^	0.38 (0.01-2.14)	2.28 (0.21-25.12)	.60^c^
TRALI	2	2 (100.0)	0.87 (0.11-3.16)	0.73 (0.56-0.94)	1.19 (0.29-4.89)	.69^c^	1.53 (0.42-3.93)	0.57 (0.10-3.11)	.69^c^

Abbreviations: DHTR, delayed hemolytic transfusion reaction; FNHTR, febrile nonhemolytic transfusion reaction; NHSN, National Healthcare Safety Network; REDS-IV-P, Recipient Epidemiology and Donor Evaluation Study-IV-Pediatric; RR, relative risk; TACO, transfusion-associated circulatory overload; TAD, transfusion-associated dyspnea; TRALI, transfusion-related acute lung injury.

^a^ Percentage of reported reactions determined to meet the definite, probable, or possible level of imputability.

^b^ χ^2^ Test used to compare REDS-IV-P reaction counts with NHSN reaction counts.

^c^ Fisher exact test used to compare REDS-IV-P reaction counts with NHSN reaction counts.

Most transfusion reactions (1070 of 1148 [93.2%]) were not severe; however, severe (60 of 1148 [5.2%]) and life-threatening (5 of 1148 [0.4%]) transfusion reactions were reported (eTable 1 in Supplement 1). There were no deaths reported during this study period that were attributable to transfusion in our patient cohort.

### Pediatric Transfusion Reaction Rate Variability by Age, Race, Hospital, and Blood Product Type

**Figure 1.**
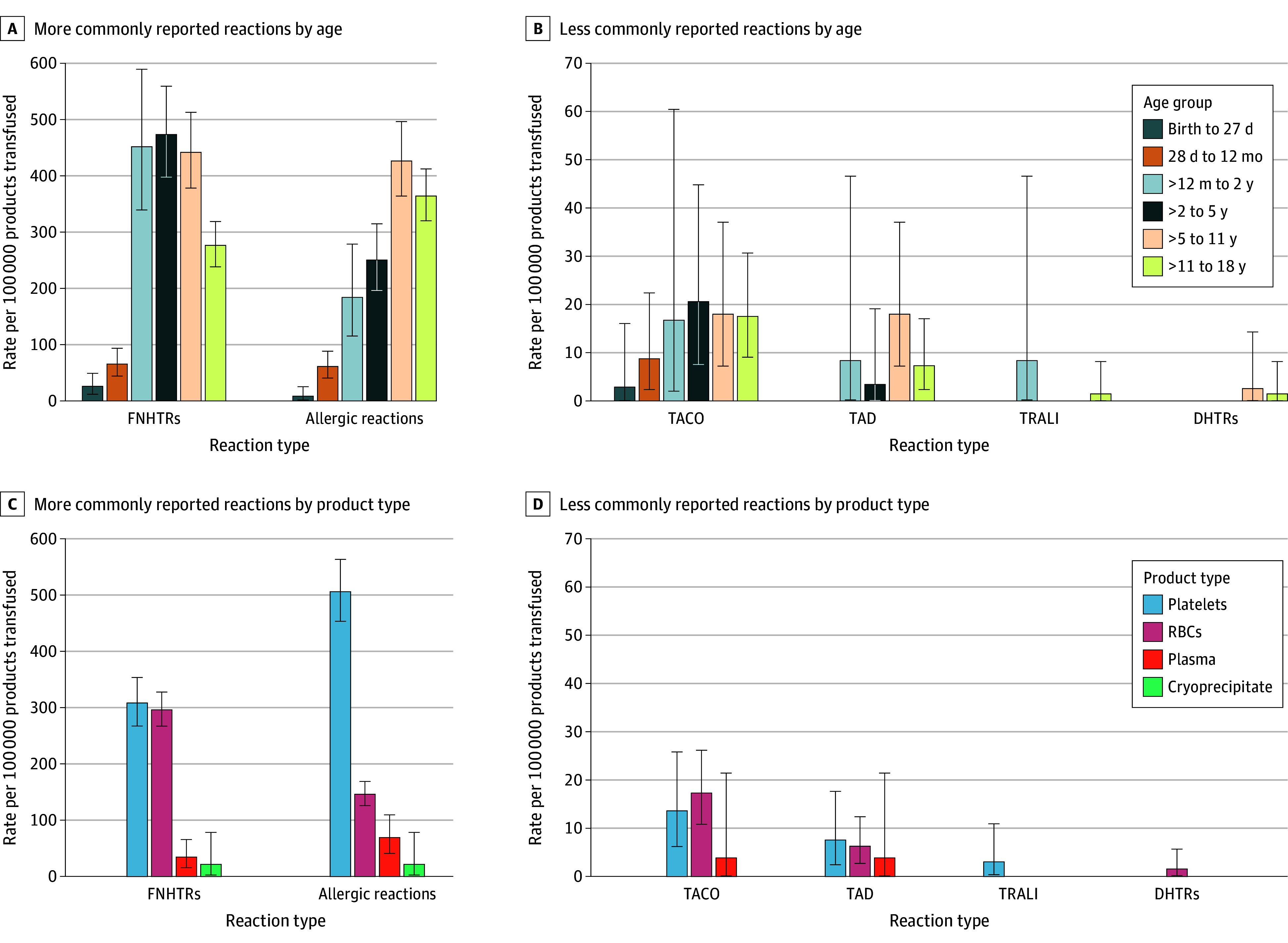
Bar Graphs of Transfusion Reaction Rate by Type, Age, and Product Transfusion reaction rates were stratified by age (A and B) and product type (C and D) for more (A, C) and less (B, D) commonly reported reactions. Error bars represent 95% CIs. DHTR indicates delayed hemolytic transfusion reaction; FNHTR, febrile nonhemolytic transfusion reaction; RBC, red blood cell; TACO, transfusion-associated circulatory overload; TAD, transfusion-associated dyspnea; TRALI, transfusion-related acute lung injury.

There were differences in transfusion reaction rates based on age, race, hospital, and blood product type in our cohort (Table 3; Figure 1). Per 100 000 products transfused, pediatric patients aged 5 to 11 years had the highest rate of transfusion reactions at 891.11 (95% CI, 799.81-989.99), while neonates from birth to 27 days old had the lowest transfusion reaction rate at 34.57 (95% CI, 17.86-60.39; *P* < .001). Transfusion reaction rates per 100 000 products transfused were similar when comparing male (497.25 [95% CI, 459.35-537.44]) and female (524.26 [95% CI, 480.53-570.89]) patients (*P* = .37). Race was a significant variable, as Asian patients had the highest rate per 100 000 products transfused of reported transfusion reactions (616.66 [95% CI, 509.11-740.20]), while Black patients had the lowest rate per 100 000 products transfused of reported transfusion reactions (398.16 [95% CI, 337.00-467.22]; *P* < .001), although transfusion reaction rates were not statistically different when stratified by ethnicity (Hispanic or Latinx vs non-Hispanic and non-Latinx). Rates also varied by hospital, with rates per 100 000 products transfused ranging from 362.15 (95% CI, 303.43-428.92) to 854.24 (95% CI, 488.27-1387.24), depending on the hospital site (*P* < .001). Transfusion reaction rates per 100 000 products transfused were significantly different depending on product type, with platelets having the highest rate (821.75 [95% CI, 754.14-893.80]) and cryoprecipitate having the lowest rate (43.33 [95% CI, 11.81-110.95]; *P* < .001).

**Table 3.** Reaction Rate by Demographic Category

**Variable**	**No. (%)**		Rate per 100 000 products transfused (95% CI)	*P* value
	Reaction	Transfusion		
Age group				
Total	1165 (100)^a^	228 886 (100)	508.99 (480.18-539.08)	<.001
0 to ≤27 d	12 (1.0)	34 708 (15.2)	34.57 (17.86-60.39)	NA
≥28 d to ≤12 mo	60 (5.2)	45 724 (20.0)	131.22 (100.14-168.91)	NA
>12 mo to ≤2 y	79 (6.8)	11 956 (5.2)	660.76 (523.13-823.50)	NA
>2 to ≤5 y	215 (18.5)	29 160 (12.7)	737.31 (642.04-842.73)	NA
>5 to ≤11 y	347 (29.8)	38 940 (17.0)	891.11 (799.81-989.99)	NA
>11 to <18 y	452 (38.8)	68 398 (29.9)	660.84 (601.31-724.66)	NA
Sex				
Total	1165 (100.0)^a^	228 886 (100.0)	508.99 (480.18-539.08)	.37
Female	529 (45.4)	100 905 (44.1)	524.26 (480.53-570.89)	NA
Male	636 (54.6)	127 903 (55.9)	497.25 (459.35-537.44)	NA
Race^b^				
Total	1165 (100.0)^a^	228 886 (100.0)	508.99 (480.18-539.08)	<.001
Asian	115 (9.9)	18 649 (8.2)	616.66 (509.11-740.20)	NA
Black	150 (12.9)	37 673 (16.5)	398.16 (337.00-467.22)	NA
White	531 (45.6)	93 824 (41.0)	565.95 (518.83-616.20)	NA
Multiracial	21 (1.8)	3680 (1.6)	570.65 (353.24-872.30)	NA
Other	333 (28.6)	68 857 (30.1)	483.61 (433.06-538.45)	NA
Unknown^c^	15 (1.3)	6203 (2.7)	NA	NA
Ethnicity				
Total	1165 (100.0)^a^	228 886 (100.0)	508.99 (480.18-539.08)	.06
Hispanic or Latinx	304 (26.1)	52 398 (22.9)	580.17 (516.78-649.20)	NA
Non-Hispanic and non-Latinx	736 (63.2)	144 017 (62.9)	511.05 (474.79-549.34)	NA
Unknown^c^	125 (10.7)	32 471 (14.2)	NA	NA
Hospital				
Total	1183 (100.0)^d^	228 886 (100.0)	516.85 (487.81-547.16)	<.001
1	376 (32.2)	72 637 (31.7)	517.64 (466.64-572.70)	NA
2	74 (6.3)	17 233 (7.5)	429.41 (337.18-539.08)	NA
3	180 (14.8)	32 574 (14.2)	552.59 (474.81-639.48)	NA
4	45 (3.9)	9147 (4.0)	491.96 (358.84-658.29)	NA
5	104 (8.8)	16 808 (7.3)	618.75 (505.57-749.72)	NA
6	254 (21.2)	41 613 (18.2)	610.39 (537.62-690.25)	NA
7	134 (11.5)	37 001 (16.2)	362.15 (303.43-428.92)	NA
8^e^	16 (1.4)	1873 (0.8)	854.24 (488.27-1387.24)	NA
Product type				
Total	1148 (100)^f^	228 886 (100)	501.56 (472.96-531.43)	<.001
Cryoprecipitate	4 (0.4)	9231 (4.0)	43.33 (11.81-110.95)	NA
Plasma	29 (2.5)	25 990 (11.4)	111.58 (74.73-160.25)	NA
Platelets	544 (47.4)	66 200 (28.9)	821.75 (754.14-893.80)	NA
Red blood cells	571 (49.7)	127 277 (55.6)	448.63 (412.58-486.98)	NA

Abbreviation: NA, not applicable.

^a^ Transfusions associated with multiple reaction types are counted as 1 event.

^b^ The multiracial classification includes individuals who self-identify as American Indian or Alaska Native and Hispanic or Latinx, Asian and Hispanic or Latinx, Black and Hispanic or Latinx, Native Hawaiian or Other Pacific Islander and Hispanic or Latinx, or other nonspecified multiethnic groups. The other classification includes individuals who self-identify as American Indian or Alaska Native, Native Hawaiian or Other Pacific Islander, or other.

^c^ Unknown includes missing values and values that were not recorded.

^d^ Transfusions associated with multiple reaction types are counted as multiple events.

^e^ Hospital 8 is a community hospital. The remaining hospitals are academic medical centers.

^f^ Transfusions associated with multiple reactions are excluded.

When stratified by age, the most common reactions for all pediatric patients were FNHTRs and allergic reactions (Figure 1A and B). There were higher rates for FNHTRs for all age ranges except for patients aged 11 to 18 years. Of the less commonly reported transfusion reactions, TACO was reported in patients of all ages. TAD, TRALI, and DHTRs were reported too infrequently to infer trends.

When stratified by product type, the most common reactions were FNHTRs and allergic reactions for all products (Figure 1C and D; eTable 2 in Supplement 1 reports absolute counts and rates). The most common transfusion reactions to RBC units were FNHTRs (296.20 [95% CI, 267.06-327.67] per 100 000 products transfused), while the most common transfusion reactions to platelets and plasma were allergic reactions (platelets, 506.04 [95% CI, 453.30-563.24] and plasma, 69.26 [95% CI, 41.05-109.46] per 100 000 products transfused). Of all platelet products transfused in the entire cohort, 23 849 units (43.5%) were pathogen reduced. Of the 544 reactions to platelet transfusions reported, 238 reactions (43.8%) occurred to pathogen-reduced platelet units. There was no observed association between pathogen reduction and reaction frequency. Four transfusion reactions to cryoprecipitate were reported, 2 FNHTRs and 2 allergic reactions. Of the less commonly reported transfusion reactions, TACO and TAD were reported after RBC (n = 30), platelet (n = 14), and plasma (n = 2) transfusions. TRALI was reported only after platelet transfusions (n = 2), and DHTRs were reported only after RBC transfusions (n = 2) (eTable 2 in Supplement 1).

### Repeat Transfusion Reaction Type

**Figure 2.**
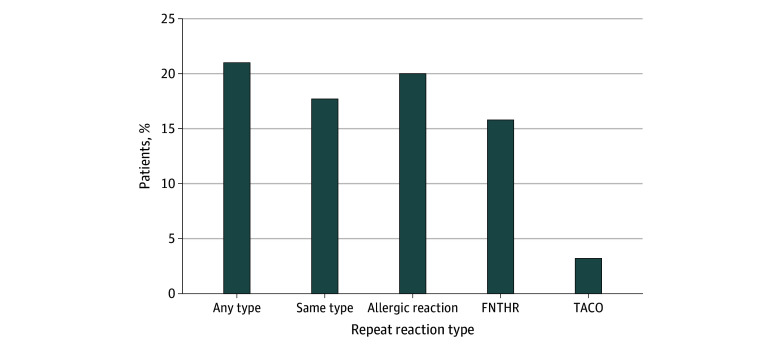
Bar Graph Summarizing Repeat Transfusion Reactions Repeat transfusion reaction rates. Among pediatric patients with documented transfusion reactions, 21.0% (185 of 866) had multiple transfusion reactions, and 85% (153 of 185) of those with multiple reactions had repeat reactions of the same type. FNHTR indicates febrile nonhemolytic transfusion reaction; TACO, transfusion-associated circulatory overload.

Many patients had multiple transfusion reactions recorded (Figure 2). During the study period, of the 866 patients in our cohort with reported reactions, 185 (21.3%) had more than 1 reaction, and the range of repeat reactions in this patient cohort was 2 to 12 reactions. Among the patients with repeat reactions, 158 (85.4%) had the same reaction type reported. Of the patients with allergic reactions, 82 (20.0% of the total number of patients with allergic reactions [n = 411]) had repeat allergic reactions. Of the patients with FNHTRs, 76 (15.8% of the total number of patients with FNHTRs [n = 481]) had repeat FNHTRs. Of the number of repeat reactions, 77.9% of repeat reactions (120 of 154) after allergic were allergic, and 72.1% of reactions (98 of 136) after FNHTRs were FNHTRs. Only 1 patient had a repeat TACO. No patients with TRALI, TAD, or DHTRs had repeat reactions of the same type.

### Most Common Symptoms Reported and Transfusion Reaction Case Definitions

As expected, the most commonly reported symptoms matched the transfusion reaction case definitions.^12^ However, for each reaction type, symptoms that were not part of standard case definition criteria were also reported (eTable 3 in Supplement 1 lists symptoms reported). Of 579 cases of FNHTRs, 96.5% (n = 559) involved fever (≥38°C), and 14.7% (n = 85) involved chills and/or rigors. The most common symptoms in the 529 cases of allergic reactions were urticaria (69.6% [n = 368]) and pruritus (30.8% [n = 163]), maculopapular rash (14.2% [n = 75]), and respiratory distress (13.2% [n = 70]). In the 24 cases of TACO, the most common signs or symptoms were acute respiratory distress (87.5% [n = 21]), evidence of fluid overload (66.7% [n = 16]), pulmonary edema (66.7% [n = 16]), biomarkers relevant to elevated brain-type natriuretic peptide or N-terminal fragment of the prohormone brain natriuretic peptide (29.2% [n = 7]), cardiovascular system changes (29.2% [n = 7]), and fever (29.2% [n = 7]). The most common symptoms in the 12 cases of TAD were acute respiratory distress (91.7% [n = 11]) and shortness of breath (58.3% [n = 7]); allergic reactions, TACO, and TRALI were ruled out in 58.3% (n = 7) of reported TAD cases. In the 2 reported DHTRs, both patients had a positive direct antiglobulin test, while 1 patient had an inadequate rise or rapid fall of posttransfusion hemoglobin, and the other patient had a newly identified RBC antibody. Lastly, in the 2 reported TRALI reactions, both patients had acute lung injury within 6 hours of transfusion, bilateral pulmonary infiltrates, hypoxemia, fever, shortness of breath, and no evidence of left atrial hypertension.

Symptoms that did not fit standard NHSN transfusion reaction case definitions were also reported. Some additional reported symptoms for 529 allergic reactions included fever (3.4% [n = 18]) and chills and/or rigors (1.9% [n = 10]). Other symptoms reported for 579 FNHTRs included nausea and/or vomiting (3.9% [n = 23]), hypotension (2.4% [n = 14]), and back pain (1.0% [n = 6]), along with the following symptoms, which occurred in fewer than 1.0% of patients: hypoxemia (0.9% [n = 5]), shortness of breath (0.9% [n = 5]), rash (0.7% [n = 4]), flank pain (0.7% [n = 4]), abdominal pain (0.5% [n = 3]), itching and/or hives (0.9% [n = 5]), cough (0.4% [n = 2]), bronchospasm (0.4% [n = 2]), jaundice (0.2% [n = 1]), and infusion site pain (0.2% [n = 1]). Additional symptoms of 24 total cases of TACO included fever (29.2% [n = 7]), hypotension (8.3% [n = 2]), and nausea and/or vomiting (8.3% [n = 2]).

### Premedication

Premedication was more likely to be administered to patients with a history of transfusion reactions, as 126 of 299 patients (42.1%) with a previous transfusion reaction during our study period received 1 or more premedication drugs before a subsequent transfusion, compared with 106 of 866 patients (12.2%) without a transfusion reaction history (*P* < .001) (eTables 4 and 5 in Supplement 1). Many patients (35.8% [107 of 299]) did not receive premedication after the first reaction in subsequent transfusions, regardless of reaction type. Among 579 patients with FNHTRs, most (53.4% [n = 309]) did not receive documented premedication. Of the 270 who did receive premedication, 5.4% (n = 31) received antipyretics, while 4.3% (n = 25) received antihistamines and antipyretics, and 4.0% (n = 23) received antihistimines. Among the 529 patients with allergic reactions, 44.4% (n = 235) did not receive premediations; 10.4% (n = 55) received antihistamines; 7.4% (n = 39) received antihistamines and antipyretics; and 4.2% (n = 23) received antihistamines, antipyretics, and steroids (eTable 6 in Supplement 1). Premedication was not commonly used for the other reported transfusion reaction types.

### Treatments and Type of Transfusion Reaction

Treatments differed based on the type of reaction reported (eTable 7 in Supplement 1). Among the 579 patients with FNHTRs, most received antipyretics (67.9% [n = 393]), while antibiotics (9.5% [n = 55]), antihistamines (6.4% [n = 37]), corticosteroids (1.0% [n = 6]), diuretics (0.3% [n = 2]), bronchodilators (0.2% [n = 1]), and inotropes and/or vasopressors (0.2% [n = 1]) were also used in some cases. The 529 patients with allergic reactions received mostly antihistamines (80.3% [n = 425]), while corticosteroids (24.0% [n = 127]), bronchodilators (7.2% [n = 38]), antipyretics (6.6% [n = 35]), inotropes and/or vasopressors (6.2% [n = 33]), antibiotics (0.8% [n = 4]), and diuretics (0.4% [n = 2]) were less commonly used. Most of the 24 patients diagnosed with TACO received diuretics (83.3% [n = 20]), while 3 patients received antipyretics (12.5%), and antibiotics (4.2%), antihistamines (4.2%), corticosteroids (4.2%), and inotropes and/or vasopressors (4.2%) were administered to 1 patient each. The 12 patients with TAD were treated with bronchodilators (33.3% [n = 4]), diuretics (33.3% [n = 4]), antihistamines (8.3% [n = 1]), antipyretics (8.3% [n = 1]), and corticosteroids (8.3% [n = 1]). Of the 2 patients with TRALI, 1 was treated with antibiotics, and the other was treated with inotropes and/or vasopressors.

## Discussion

In this multicenter cohort study, pediatric patients experienced significantly higher rates of transfusion reactions compared with all-age populations reported through the NHSN Hemovigilance Module. These differences were attributable to increased rates of allergic reactions and FNHTRs, while respiratory reactions, including TACO, TAD, and TRALI, were rarely reported, and rates were similar to national all-age data. Although prior publications have reported higher rates of allergic reactions and FNHTRs in pediatric populations,^3,4^ our findings provide standardized insights into the epidemiology of pediatric transfusion reactions in the US due to the granularity of the data we prospectively collected from 8 hospitals across the US.

Reaction rates varied widely across study sites, which may reflect differences in patient populations, underlying disease prevalence, transfusion practices, or local approaches to transfusion reaction recognition and reporting. Similar variability has been reported in prior hemovigilance studies,^15,16^ underscoring the influence of local culture and reporting standards on apparent incidence. Differences by age and race were also observed, with neonates having the lowest reaction rates and school-aged children the highest and with Asian patients experiencing higher reaction rates compared with Black patients. These findings suggest that biological and social factors and practice-related contributors may influence susceptibility and recognition of transfusion reactions in children. As an example, the paucity of transfusion reactions reported in neonates may either represent the true low transfusion reaction rate in neonates due to their immature immune system function or the difficulty in recognizing transfusion reactions in neonates because symptoms overlap with other neonatal pathologies. Additionally, higher rates of allergic and febrile reactions in school-aged children are likely driven by heightened immune system reactivity in this cohort compared with neonates. Indeed, other groups have reported that a patient’s own underlying susceptibility to allergies is associated with a risk of allergic transfusion reactions in both adult and pediatric populations.^17,18^

Although this dataset was large, the number of reported respiratory reactions was expectedly low.^19^ Respiratory complications are known to be underrecognized in both pediatric and adult settings,^19,20^ particularly when symptoms overlap with infection, fluid overload, or baseline respiratory compromise. Nearly one-third of pediatric TACO cases also presented with fever, underscoring the diagnostic complexity and a potential novel diagnostic feature of TACO in children. Although fever and inflammatory responses have been reported in almost one-third to two-thirds of patients presenting with TACO,^21,22^ this phenomenon has not been reported in a pediatric-specific cohort. The underlying mechanism for fever in TACO remains poorly understood. This finding highlights the need for improved diagnostic criteria and surveillance methods for respiratory transfusion reactions in children.

A key strength of this study is the linking of detailed data on symptoms, premedication, and treatments with multicenter, vein-to-vein data, yielding a complete view of the presentation and management of pediatric transfusion reactions. Most reactions aligned with case definitions, although nonclassical features were also observed. Premedication use was inconsistent, even among patients with prior reactions, reflecting the lack of consensus in this area. Conclusions from a systematic review and meta-analysis suggest that routine prophylaxis with acetaminophen or antihistamines does not prevent FNHTRs or allergic reactions.^23^ Across institutions, premedication use is often applied inconsistently, and a recent quality improvement initiative showed that standardizing premedication policies reduced unnecessary prophylaxis without increasing reaction rates.^24^ Additionally, not all patients with prior reactions benefit equally from prophylaxis, as children with a history of mild allergic transfusion reactions do not need premedication for subsequent transfusions,^25^ whereas patients with a history of severe allergic reactions may benefit from premedication.^26^ Together, these findings indicate that while universal premedication is not supported by evidence, targeted use in patients with prior severe reactions may be beneficial, highlighting the need for prospective pediatric studies to define evidence-based prophylaxis strategies.

This study also has important hemovigilance implications. To our knowledge, this was the first study to use a US multicenter dataset dedicated to pediatric transfusion reactions, providing a benchmark for institutions and a foundation for future reporting. Current national hemovigilance systems, including the NHSN, do not stratify pediatric data, limiting the ability to monitor age-specific risks. Our findings underscore the need for pediatric-specific reporting modules or more pediatric-specific standards to improve pediatric transfusion reaction investigations. With the observed site-level variability, standardized recognition and reporting practices will be critical to ensure consistency and comparability across institutions.

### Limitations

Several study limitations should be acknowledged. As with any surveillance-based study, underrecognition and underreporting, particularly of respiratory events, are likely. In addition, we do not have specific hospital information for the NHSN all-ages or pediatric transfusion reaction datasets,^4,7^ limiting a more direct comparison with our cohort, as most transfusion reactions from these studies were gathered from the NHSN or the Association for the Advancement of Blood and Biotherapies Center for Patient Safety database. Premedication data were missing in nearly one-third of cases, and we could not determine whether patients who received premedication during the study period had transfusion reactions prior to the data collection time frame, which may have influenced clinical decisions. Finally, institutional variability suggests that some observed differences may reflect reporting practices rather than true biological variation.

## Conclusions

In this cohort study of pediatric transfusion reaction rates, pediatric patients had disproportionately higher rates of FNHTRs and allergic reactions compared with adults, while rates of respiratory and hemolytic reactions were similar. This study provides the first standardized, multicenter evaluation of pediatric transfusion reactions in the US and establishes an important benchmark for future surveillance. Beyond quantifying reaction rates, our findings underscore the role of institutional variability, the inconsistency of premedication practices, and the tendency for repeated same-type reactions, all of which have implications for clinical management and hemovigilance. Future studies should define risk factors for specific reaction types, investigate biological and clinical factors in repeat reactions, clarify the role of premedication strategies in children, and ensure that pediatric transfusion reactions are stratified by age and reported separately in national surveillance systems. Such efforts are essential to guide evidence-based practice and improve transfusion safety for children.

## Notes

Supplement 1. **eFigure.** Example of Standardized Transfusion Reaction Data Collection Forms **eTable 1.** Severity of Imputable REDS-IV-P Transfusion Reactions by Reaction Type **eTable 2.** Reaction Count & Rate Stratified by Product Type and Reaction Rate **eTable 3.** Symptom Summary Table **eTable 4.** Pre-Medication Frequency by History of Reaction **eTable 5.** Pre-Medications for Patients with a Previous Reaction **eTable 6.** Pre-Medications for Patients with a Transfusion Reaction **eTable 7.** Treatments Provided Post-Transfusion Reaction

Supplement 2.
Data Sharing Statement
