# Supplementary material for: Epidemiology of Pediatric Transfusion Reactions
Source: JAMA Netw Open. 2026 Apr 27;9(4):e269274. doi: 10.1001/jamanetworkopen.2026.9274 (PMC13122406; doi:10.1001/jamanetworkopen.2026.9274)
Supplement: Supplement 1. — eFigure. Example of Standardized Transfusion Reaction Data Collection Forms eTable 1. Severity of Imputable REDS-IV-P Transfusion Reactions by Reaction Type eTable 2. Reaction Count & Rate Stratified by Product Type and Reaction Rate eTable 3. Symptom Summary Table eTable 4. Pre-Medication Frequency by History of Reaction eTable 5. Pre-Medications for Patients with a Previous Reaction eTable 6. Pre-Medications for Patients with a Transfusion Reaction eTable 7. Treatments Provided Post-Transfusion Reaction [file jamanetwopen-e269274-s001.pdf]

Supplemental Online Content

Stone EF, Chacreton D, Jimenez A, et al. Epidemiology of pediatric transfusion reactions. *JAMA Netw Open*. 2026;9(4):e269274. doi:10.1001/jamanetworkopen.2026.9274

eFigure 1. Example of Standardized Transfusion Reaction Data Collection Forms

eTable 1. Severity of Imputable REDS-IV-P Transfusion Reactions by Reaction Type

eTable 2. Reaction Count & Rate Stratified by Product Type and Reaction Rate

eTable 3. Symptom Summary Table

eTable 4. Pre-Medication Frequency by History of Reaction

eTable 5. Pre-Medications for Patients with a Previous Reaction

eTable 6. Pre-Medications for Patients with a Transfusion Reaction

eTable 7. Treatments Provided Post-Transfusion Reaction

This supplemental material has been provided by the authors to give readers additional information about their work.

eFigure 1. **Example of standardized transfusion reaction data collection forms.** A. The specific form for reporting all reactions (Pediatric Transfusion Reactions), and B. Febrile nonhemolytic transfusion reactions (FNHTR) as an example.
A.

Pediatric Transfusion Reactions*
(Patients <18 years of age)

**** Complete all information in this form***

Pediatric Transfusion Reactions Lookup

To create or edit the Pediatric Transfusion Reactions forms, please enter the Donation Identification Number (DIN) and Product Code (including division code) for the potential implicated unit, or if many, the DIN and Product Code for the unit received by patient that is closest in time to the reaction in question. This will be the only link to V2V data.

If the transfused unit was an aliquot or split product, be sure to include the correct division code in the seventh and eighth characters of the Product Code (e.g. E0332VAb).

DIN* ___________________________ (character string length 13)

Product Code (including division code)* ___________________ (character string length 8)

Component Details

The Donation Identification number (DIN) and Product Code together uniquely identify a unit/aliquot transfused and are both required to link to the correct patient in V2V data. Make sure they are correct. Go back to the Pediatric Transfusion Reactions Lookup page to enter a different DIN and Product Code for a different set of forms.

DIN* ___________________________ (character string length 13) *[programming note: pre-populated from Lookup page]*

Product Code (including division code)* ___________________ (character string length 8) *[programming note: pre-populated from Lookup page]*

Are there other potential implicated units?*
- ☐ No
- ☐ Yes*

**If yes, make sure the DIN and Product Code recorded above is for the unit closest in time to the reaction in question.*

Reaction Type (check all that apply)*

Please refer to the CDC NHSN Hemovigilance Module Surveillance Protocol to determine which reaction type(s) are applicable.

- ☐ TRALI
- ☐ TACO
- ☐ Transfusion Associated Dyspnea
- ☐ Febrile Non-Hemolytic
- ☐ Delayed Hemolytic
- ☐ Allergic
- ☐ Other Reaction Type

eFigure 1 (continued). B.

Adapted for Joint Program in Transfusion Medicine (JPTM) from: https://www.cdc.gov/nhsn/acute-care-hospital/bio-hemo/
Updated: 12/10/17

NHSN Additional Reaction Data Form: Febrile Non-Hemolytic Transfusion Reaction*

*** Complete all information in this form***

Reaction Details

Reaction Details		
Date reaction occurred: ____/____/____ (MM/DD/YYYY)	Time reaction occurred: __ __:__ __ (24-hour clock)	☐ Reaction time unknown
	Transfusion start date: ____/____/____ (MM/DD/YYYY)	
	Transfusion start time: __ __:__ __ (24-hour clock)	☐ Start time unknown
	Transfusion end date: ____/____/____ (MM/DD/YYYY)	
	Transfusion end time: __ __:__ __ (24-hour clock)	☐ End time unknown
	Total volume transfused (mL): ____ (enter whole number, range: 0-600mL)	☐ Volume unknown

Investigation Results

Febrile non-hemolytic transfusion reaction (FNHTR)

Case Definition:

Check all that occurred during or within **4 hours** of cessation of transfusion (check all that apply):

- ☐ Fever (greater than or equal to 38°C/100.4°F oral and a change of at least 1°C/1.8°F) from pre-transfusion value
- ☐ Chills/rigors are present

Other signs and symptoms (check all that apply):

Generalized:	☐ Nausea/vomiting			
Cardiovascular:	☐ Blood pressure decrease	☐ Shock		
Cutaneous:	☐ Edema	☐ Flushing	☐ Jaundice	
	☐ Pruritus (itching)	☐ Urticaria (hives)	☐ Other rash	
Hemolysis/Hemorrhage:	☐ Disseminated intravascular coagulation	☐ Hemoglobinemia		
	☐ Positive antibody screen			
Pain:	☐ Abdominal pain	☐ Back pain	☐ Flank pain	☐ Infusion site pain
Renal:	☐ Hematuria	☐ Hemoglobinuria	☐ Oliguria	
Respiratory:	☐ Bilateral infiltrates on chest x-ray	☐ Bronchospasm	☐ Cough	
	☐ Hypoxemia	☐ Shortness of breath		

☐ Other sign/symptom

☐ No signs/symptoms (single-select)

Continued >>

© 2026 Stone EF, et al. *JAMA Network Open*.

eFigure 1.B. (continued)

Adapted for Joint Program in Transfusion Medicine (JPTM) from: https://www.cdc.gov/nhsn/acute-care-hospital/bio-hemo/
Updated: 12/10/17

REDS-IV-P

Select all the cultures that were obtained for the **blood bag in proximity** to transfusion febrile reaction (check all that apply):

☐ Blood culture (specify result below)
  ☐ Negative
  ☐ Positive; specify organism(s): ____________________

☐ Fungal culture (specify result below)
  ☐ Negative
  ☐ Positive; specify organism(s): ____________________

☐ Viral culture (specify result below)
  ☐ Negative
  ☐ Positive; specify organism(s): ____________________

☐ None of the above (single-select)

Severity – Indicate the severity of the reaction (select only one)**:**

☐ Non-severe (symptomatic treatment only)

.

☐ Severe (hospitalization, including prolonged hospitalization, disability, and/or incapacitation)

☐ Life-threatening

☐ Death

☐ Not determined

Imputability (select only one):

Which best describes the relationship between the transfusion and the reaction?

☐ Definite (Patient has no other conditions that could explain signs/symptoms.)

☐ Probable (There are other potential causes present that could explain signs/symptoms, but transfusion is the most likely cause.)

☐ Possible (Other present causes are most likely, but transfusion cannot be ruled out.)

☐ Doubtful (Evidence is clearly in favor of a cause other than the transfusion, but transfusion cannot be excluded.)

☐ Ruled out (There is conclusive evidence beyond reasonable doubt of a cause other than the transfusion.)

☐ Not determined (The relationship between the adverse reaction and the transfusion is unknown or not stated.)

Continued >>

eFigure 1.B. (continued)

CDC NHSN Criteria

Please refer to the CDC NHSN Hemovigilance Module Surveillance Protocol to complete the below questions. Select one for each:

CDC NHSN Case Definition:

- ☐ Definitive
- ☐ Possible

CDC NHSN Severity:*

**Automatically selected if the same response was previously selected in the Severity section above.*

- ☐ Non-severe
- ☐ Severe
- ☐ Life-threatening*
- ☐ Death*
- ☐ Not determined

CDC NHSN Imputability:*

**Automatically selected if the same response was previously selected in the Imputability section above.*

- ☐ Definite
- ☐ Probable
- ☐ Possible
- ☐ Doubtful
- ☐ Ruled out
- ☐ Not determined

Outcome (select only one)

Outcome:	☐ Not determined	☐ Minor or no sequelae	☐ Major or long-term sequelae	☐ Death*
				**Automatically selected if death is selected in the Severity section above. Additional details warranted for this Outcome.*<br><br>Date of death: ____/____/____ (MM/DD/YYYY)<br><br>Relationship of transfusion to death: (Select one)<br>☐ Definite<br>☐ Probable<br>☐ Possible<br>☐ Doubtful<br>☐ Ruled out<br>☐ Not determined

Continued >>

© 2026 Stone EF, et al. *JAMA Network Open*.

eFigure 1.B. (continued)

Adapted for Joint Program in Transfusion Medicine (JPTM) from: https://www.cdc.gov/nhsn/acute-care-hospital/bio-hemo/
Updated: 12/10/17

Patient Treatment

Did the patient receive prophylactic treatment **prior to** the transfusion (premedication)?

☐ NO (skip to next question) ☐ YES, continue below ☐ UNKNOWN (skip to next question)

If yes, select treatment(s) (check all that apply) :

- ☐ Antihistamine (H1 or H2 blocker)
- ☐ Antipyretic
- ☐ Steroid
- ☐ Other

Did the patient receive treatment for the transfusion reaction? ☐ NO (done) ☐ YES, continue below

If yes, select treatment(s) (check all that apply):

- ☐ **Medication** *(check all that apply)*
  - ☐ Antipyretics
  - ☐ Antihistamines (H1 or H2 blockers)
  - ☐ Inotropes/Vasopressors
  - ☐ Bronchodilator
  - ☐ Diuretics
  - ☐ Intravenous Immunoglobulin
  - ☐ Corticosteroids
  - ☐ Antibiotics
  - ☐ Antithymocyte globulin
  - ☐ Cyclosporin
  - ☐ Other
- ☐ **Volume resuscitation** (Intravenous colloids or crystalloids)
- ☐ **Respiratory support** *(check all that apply)*
  - ☐ Mechanical ventilation
  - ☐ Noninvasive ventilation
  - ☐ Oxygen
  - ☐ Other
- ☐ **Renal replacement therapy** *(check all that apply)*
  - ☐ Hemodialysis
  - ☐ Peritoneal dialysis
  - ☐ Continuous Veno-Venous Hemofiltration
  - ☐ Other
- ☐ **Phlebotomy**
- ☐ **Other treatment**

Adapted for JPTM on 2017.01.20 using CDC 57.311 R0, v8.6 Page 4 of 4

© 2026 Stone EF, et al. *JAMA Network Open*.

eTable 1. Severity of Imputable REDS-IV-P Transfusion Reactions by Reaction Type

Transfusion Reaction	Non-severe Rxn, n (%)	Severe Rxn, n (%)	Life-threatening Rxn, n (%)	Not Determined Rxn, n (%)	Total Rxn, n (%)
Allergic	477 (90.20)	48 (9.10)	1 (0.20)	3 (0.60)	529 (100.00)
DHTR	0 (0.00)	1 (50.00)	0 (0.00)	1 (50.00)	2 (100.00)
TAD	11 (91.70)	1 (8.30)	0 (0.00)	0 (0.00)	12 (100.00)
FNHTR	565 (97.60)	8 (1.40)	0 (0.00)	6 (1.00)	579 (100.00)
TACO	17 (70.80)	2 (8.30)	3 (12.50)	2 (8.30)	24 (100.00)
TRALI	0 (0.00)	0 (0.00)	1 (50.00)	1 (50.00)	2 (100.00)
Total	1,070 (93.21)	60 (5.23)	5 (0.44)	13 (1.13)	1,148 (100.00)

Note. Transfusions with multiple reactions excluded.

© 2026 Stone EF, et al. *JAMA Network Open*.

eTable 2. Reaction Count & Rate Stratified by Product Type and Reaction Rate

Grouping	Total n (%)	Total Rate per 100k (95% CI)	Red Blood Cells, n (%)	Red Blood Cells Rate per 100k (95% CI)	Platelets, n (%)	Platelets Rate per 100k (95% CI)
Transfusion Counts	228,886 (100.0)	-	127,277 (55.61)	-	66,200 (28.92)	-
Overall Reaction	1,183 (100.0)	516.85 (487.81, 547.16)	595 (50.04)	467.48 (430.67, 506.60)	555 (47.12)	838.37 (770.06, 911.11)
FNHTR Reaction	592 (100.0)	258.64 (238.23, 280.34)	377 (63.68)	296.20 (267.06, 327.67)	204 (34.46)	308.16 (267.32, 353.47)
Allergic Reaction	541 (100.0)	236.36 (216.86, 257.15)	186 (34.38)	146.14 (125.89, 168.72)	335 (61.92)	506.04 (453.30, 563.24)
TACO Reaction	32 (100.0)	13.98 (9.56, 19.74)	22 (68.75)	17.29 (10.83, 26.17)	9 (28.12)	13.60 (6.22, 25.81)
TAD Reaction	14 (100.0)	6.12 (3.34, 10.26)	8 (57.14)	6.29 (2.71, 12.38)	5 (35.71)	7.55 (2.45, 17.63)
DHTR Reaction	2 (100.0)	0.87 (0.11, 3.16)	2 (100.0)	1.57 (0.19, 5.68)	-	-
TRALI Reaction	2 (100.0)	0.87 (0.11, 3.16)	-	-	2 (100.0)	3.02 (0.37, 10.91)

eTable 2. Reaction Count & Rate Stratified by Product Type and Reaction Rate (cont.)

Grouping	Plasma, n (%)	Plasma Rate per 100k (95% CI)	Cryo, n (%)	Cryo Rate per 100k (95% CI)
Transfusion Counts	25,990 (11.35)	-	9,231 (4.03)	-
Overall Reaction	29 (2.49)	111.58 (74.73, 160.25)	4 (0.34)	43.33 (11.81, 110.95)
FNHTR Reaction	9 (1.52)	34.63 (15.83, 65.74)	2 (0.34)	21.67 (2.62, 78.27)
Allergic Reaction	18 (3.33)	69.26 (41.05, 109.46)	2 (0.37)	21.67 (2.62, 78.27)
TACO Reaction	1 (3.12)	3.85 (0.10, 21.44)	-	-
TAD Reaction	1 (7.14)	3.85 (0.10, 21.44)	-	-
DHTR Reaction	-	-	-	-
TRALI Reaction	-	-	-	-

© 2026 Stone EF, et al. *JAMA Network Open*.

eTable 3. Symptom Summary Table

Sign/Symptom	FNHTR Reaction n=579 [Symptom n, (%)]	Allergic Reaction n=529 [Symptom n, (%)]	TACO Reaction n=24 [Symptom n, (%)]	TAD Reaction n=12 [Symptom n, (%)]	TRALI Reaction n=2 [Symptom n, (%)]	DHTR Reaction n=2 [Symptom n, (%)]
ALI w/in 6-hours of Transfusion	-	-	-	-	2 (100.00)[a]	-
Abdominal pain	3 (0.52)	5 (0.95)	0 (0.00)	0 (0.00)	0 (0.00)	3 (0.52)
Acute respiratory distress	-	-	21 (87.50)[a]	11 (91.67)[a]	-	-
Allergic reaction, TACO, and TRALI definitions are not applicable	-	-	-	7 (58.33)[a]	-	-
Angioedema	-	24 (4.54)[a]	-	-	-	-
Back pain	6 (1.04)	3 (0.57)	0 (0.00)	0 (0.00)	0 (0.00)	6 (1.04)
Bilateral infiltrates	0 (0.00)	0 (0.00)	-	0 (0.00)	2 (100.00)[a]	0 (0.00)
Bronchospasm	2 (0.35)	-	0 (0.00)	2 (16.67)	0 (0.00)	2 (0.35)
Cardiovascular shock	0 (0.00)	0 (0.00)	0 (0.00)	0 (0.00)	1 (50.00)	0 (0.00)
Cardiovascular system changes	-	-	7 (29.17)[a]	-	-	-
Chills/rigors	85 (14.68)[a]	10 (1.89)	0 (0.00)	0 (0.00)	1 (50.00)	85 (14.68)[a]
Conjunctival edema	-	10 (1.89)[a]	-	-	-	-
Cough	2 (0.35)	59 (11.15)	1 (4.17)	2 (16.67)	0 (0.00)	2 (0.35)
Distress or bronchospasm	-	70 (13.23)[a]	-	-	-	-
Edema	0 (0.00)	-	3 (12.50)	0 (0.00)	0 (0.00)	0 (0.00)
Edema of lips, tongue, and uvula	-	32 (6.05)[a]	-	-	-	-
Elevated brain natriuretic peptide (BNP) or NT-pro BNP relevant biomarker	-	-	7 (29.17)[a]	-	-	-
Evidence of fluid overload	-	-	16 (66.67)[a]	-	-	-
Fever (greater than or equal to 38°C/100.4°F)	559 (96.55)[a]	18 (3.40)	7 (29.17)	0 (0.00)	2 (100.00)	559 (96.55)[a]
Flank pain	4 (0.69)	3 (0.57)	0 (0.00)	0 (0.00)	0 (0.00)	4 (0.69)
Generalized flushing	14 (2.42)	43 (8.13)[a]	1 (4.17)	0 (0.00)	0 (0.00)	14 (2.42)
Hemoglobinuria	0 (0.00)	1 (0.19)	0 (0.00)	0 (0.00)	0 (0.00)	0 (0.00)
Hemolysis/hemorrhage disseminated intravascular coagulation	0 (0.00)	0 (0.00)	0 (0.00)	0 (0.00)	0 (0.00)	0 (0.00)
Hemolysis/hemorrhage hemoglobinemia	0 (0.00)	0 (0.00)	0 (0.00)	0 (0.00)	0 (0.00)	0 (0.00)
Hemolysis/hemorrhage positive antibody screen	0 (0.00)	0 (0.00)	0 (0.00)	0 (0.00)	0 (0.00)	0 (0.00)
Hypotension	14 (2.42)	26 (4.91)[a]	2 (8.33)	0 (0.00)	1 (50.00)	14 (2.42)
Hypoxemia	5 (0.86)	9 (1.70)	-	4 (33.33)	2 (100.00)[a]	5 (0.86)

eTable 3. Symptom Summary Table (continued)

Sign/Symptom	FNHTR Reaction n=579 [Symptom n, (%)]	Allergic Reaction n=529 [Symptom n, (%)]	TACO Reaction n=24 [Symptom n, (%)]	TAD Reaction n=12 [Symptom n, (%)]	TRALI Reaction n=2 [Symptom n, (%)]	DHTR Reaction n=2 [Symptom n, (%)]
Inadequate rise or rapid fall of post-transfusion hemoglobin level	-	-	-	-	-	-
Infusion site pain	1 (0.17)	1 (0.19)	0 (0.00)	0 (0.00)	0 (0.00)	1 (0.17)
Jaundice	1 (0.17)	0 (0.00)	0 (0.00)	0 (0.00)	0 (0.00)	1 (0.17)
Maculopapular rash	4 (0.69)	75 (14.18)[a]	0 (0.00)	0 (0.00)	0 (0.00)	4 (0.69)
Nausea/vomiting	23 (3.97)	34 (6.43)	2 (8.33)	0 (0.00)	0 (0.00)	23 (3.97)
Newly-identified red blood cell alloantibody	-	-	-	-	-	-
No Prior ALI	-	-	-	-	0 (0.00)[a]	-
No evidence of left atrial hypertension	-	-	-	-	2 (100.00)[a]	-
Other/unspecified symptom	66 (11.40)	90 (17.01)	9 (37.50)	1 (8.33)	1 (50.00)	66 (11.40)
Otherwise unexplained appearance of spherocytes	-	-	-	-	-	-
Periorbital edema/erythema	-	40 (7.56)[a]	-	-	-	-
Positive direct antiglobulin test (DAT)	-	-	-	-	-	-
Positive elution test with alloantibody	-	-	-	-	-	-
Pruritus (itching)	3 (0.52)	163 (30.81)[a]	0 (0.00)	0 (0.00)	0 (0.00)	3 (0.52)
Pulmonary edema	-	-	16 (66.67)[a]	-	-	-
Renal hematuria	0 (0.00)	0 (0.00)	0 (0.00)	0 (0.00)	0 (0.00)	0 (0.00)
Renal oliguria	0 (0.00)	0 (0.00)	0 (0.00)	0 (0.00)	0 (0.00)	0 (0.00)
Shortness of breath	5 (0.86)	34 (6.43)	5 (20.83)	7 (58.33)	2 (100.00)	5 (0.86)
Urticaria (hives)	2 (0.35)	368 (69.57)[a]	0 (0.00)	0 (0.00)	0 (0.00)	2 (0.35)

Note. Transfusions with multiple reactions excluded. [a] = Symptom included in transfusion reaction case definition. - = Symptom not associated with reaction.

© 2026 Stone EF, et al. *JAMA Network Open*.

eTable 4. Pre-Medication Frequency by History of Reaction

Pre-Medication Given	Reaction History, n (%)	No Reaction History, n (%)	*P* Value
Yes	126 (42.14)	106 (12.24)	<0.001
No	107 (35.79)	466 (53.81)	-
Unknown	66 (22.07)	294 (33.95)	-
Note. Chi-squared test used for comparison.			

© 2026 Stone EF, et al. *JAMA Network Open*.

eTable 5. Pre-Medications for Patients with a Previous Reaction

Pre-Medication Given	Total, n (%)	FNHTR, n (%)	Allergic, n (%)	TAD, n (%)	TACO, n (%)	TRALI, n (%)	DHTR, n (%)
None Given	105 (35.84)	65 (50.00)	39 (24.84)	-	1 (25.00)	-	-
Unknown	64 (21.84)	25 (19.23)	38 (24.20)	-	1 (25.00)	-	-
Antihistamines	42 (14.33)	12 (9.23)	30 (19.11)	-	-	-	-
Antihistamines & Antipyretics	37 (12.63)	16 (12.31)	21 (13.38)	-	-	-	-
Antihistamines, Antipyretics, & Steroids	21 (7.17)	1 (0.77)	18 (11.46)	1 (50.00)	1 (25.00)	-	-
Antipyretics	14 (4.78)	10 (7.69)	2 (1.27)	1 (50.00)	1 (25.00)	-	-
Antihistamines & Steroids	4 (1.37)	1 (0.77)	3 (1.91)	-	-	-	-
Antihistamines, Antipyretics, Steroids, & Other	2 (0.68)	-	2 (1.27)	-	-	-	-
Antihistamines, Steroids & Other	2 (0.68)	-	2 (1.27)	-	-	-	-
Antihistamines & Other	1 (0.34)	-	1 (0.64)	-	-	-	-
Antipyretics & Steroids	1 (0.34)	-	1 (0.64)	-	-	-	-

Note. Transfusions with multiple reactions excluded.

eTable 6. Pre-Medications for Patients with a Transfusion Reaction

Pre-Medication Given	Total, n (%)	FNHTR, n (%)	Allergic, n (%)	TAD, n (%)	TACO, n (%)	TRALI, n (%)	DHTR, n (%)
None Given	567 (49.39)	309 (53.37)	235 (44.42)	8 (66.67)	12 (50.00)	2 (100.00)	1 (50.00)
Unknown	351 (30.57)	188 (32.47)	156 (29.49)	1 (8.33)	5 (20.83)	-	1 (50.00)
Antihistamines	78 (6.79)	23 (3.97)	55 (10.40)	-	-	-	-
Antihistamines & Antipyretics	66 (5.75)	25 (4.32)	39 (7.37)	1 (8.33)	1 (4.17)	-	-
Antipyretics	42 (3.66)	31 (5.35)	8 (1.51)	1 (8.33)	2 (8.33)	-	-
Antihistamines, Antipyretics, & Steroids	25 (2.18)	1 (0.17)	22 (4.16)	1 (8.33)	1 (4.17)	-	-
Antihistamines & Steroids	7 (0.61)	2 (0.35)	5 (0.95)	-	-	-	-
Other	4 (0.35)	-	2 (0.38)	-	2 (8.33)	-	-
Antihistamines, Antipyretics, Steroids, & Other	3 (0.26)	-	2 (0.38)	-	1 (4.17)	-	-
Antihistamines, Steroids & Other	3 (0.26)	-	3 (0.57)	-	-	-	-
Antihistamines & Other	1 (0.09)	-	1 (0.19)	-	-	-	-

Note. Transfusions with multiple reactions excluded.

eTable 7. Treatments Provided Post-Transfusion Reaction

Treatment	FNHTR Reaction, n=579 [Treatment n, (%)]	Allergic Reaction, n=529 [Treatment n, (%)]	TACO Reaction, n=24 [Treatment n, (%)]	TAD Reaction, n=12 [Treatment n, (%)]	TRALI Reaction, n=2 [Treatment n, (%)]
Antibiotics	55 (9.50)	4 (0.76)	1 (4.17)	0 (0.00)	1 (50.00)
Antihistamines (H1 or H2 blockers)	37 (6.39)	425 (80.34)	1 (4.17)	1 (8.33)	0 (0.00)
Antipyretics	393 (67.88)	35 (6.62)	3 (12.50)	1 (8.33)	0 (0.00)
Bronchodilator	1 (0.17)	38 (7.18)	0 (0.00)	4 (33.33)	0 (0.00)
Corticosteroids	6 (1.04)	127 (24.01)	1 (4.17)	1 (8.33)	0 (0.00)
Diuretics	2 (0.35)	2 (0.38)	20 (83.33)	4 (33.33)	0 (0.00)
Inotropes/Vasopressors	1 (0.17)	33 (6.24)	1 (4.17)	0 (0.00)	1 (50.00)
Other	11 (1.90)	27 (5.10)	3 (12.50)	0 (0.00)	0 (0.00)
Note. Transfusions with multiple reactions excluded.					
