# Supplementary material for: Epidemiology of Pediatric Transfusion Reactions
Source: JAMA Netw Open. 2026 Apr 27;9(4):e269274. doi: 10.1001/jamanetworkopen.2026.9274 (PMC13122406; doi:10.1001/jamanetworkopen.2026.9274)
Supplement: Supplement 2. — Data Sharing Statement [file jamanetwopen-e269274-s002.pdf]

Data Sharing Statement

Stone. Epidemiology of Pediatric Transfusion Reactions. *JAMA Netw Open*. Published April 27, 2026. doi:10.1001/jamanetworkopen.2026.9274

Data
Data available: No

Additional Information
Explanation for why data not available: The public use files that will be available at a future date will include the Vein-To-Vein databases, which include patient, donor, and component data. Sub-study data, including transfusion reaction data collection or transfusion reaction analysis data, will not be made publicly available.
